# Andean Condor (*Vultur gryphus*) in Ecuador: Geographic Distribution, Population Size and Extinction Risk

**DOI:** 10.1371/journal.pone.0151827

**Published:** 2016-03-17

**Authors:** Adrián Naveda-Rodríguez, Félix Hernán Vargas, Sebastián Kohn, Galo Zapata-Ríos

**Affiliations:** 1 Wildlife Conservation Society Ecuador Program, Quito, Pichincha, Ecuador; 2 The Peregrine Fund, Boise, Idaho, United States of America; 3 Centro de Rescate Ilitío, Quito, Pichincha, Ecuador; 4 Grupo Nacional de Trabajo del Cóndor Andino en Ecuador, Quito, Pichincha, Ecuador; Sichuan University, CHINA

## Abstract

The Andean Condor (*Vultur gryphus*) in Ecuador is classified as Critically Endangered. Before 2015, standardized and systematic estimates of geographic distribution, population size and structure were not available for this species, hampering the assessment of its current status and hindering the design and implementation of effective conservation actions. In this study, we performed the first quantitative assessment of geographic distribution, population size and population viability of Andean Condor in Ecuador. We used a methodological approach that included an ecological niche model to study geographic distribution, a simultaneous survey of 70 roosting sites to estimate population size and a population viability analysis (PVA) for the next 100 years. Geographic distribution in the form of extent of occurrence was 49 725 km^2^. During a two-day census, 93 Andean Condors were recorded and a population of 94 to 102 individuals was estimated. In this population, adult-to-immature ratio was 1:0.5. In the modeled PVA scenarios, the probability of extinction, mean time to extinction and minimum population size varied from zero to 100%, 63 years and 193 individuals, respectively. Habitat loss is the greatest threat to the conservation of Andean Condor populations in Ecuador. Population size reduction in scenarios that included habitat loss began within the first 15 years of this threat. Population reinforcement had no effects on the recovery of Andean Condor populations given the current status of the species in Ecuador. The population size estimate presented in this study is the lower than those reported previously in other countries where the species occur. The inferences derived from the population viability analysis have implications for Condor management in Ecuador. This study highlights the need to redirect efforts from captive breeding and population reinforcement to habitat conservation.

## Introduction

Andean Condor (*Vultur gryphus*), the largest neotropical scavenger in South America, is distributed between the latitudes 11° N and 55° S, from northern Colombia and Western Venezuela through the Andes in Ecuador, Peru and Bolivia to Tierra del Fuego in Argentina and Chile, descending to sea level in Chile and Peru [1, 2]. Like other tropical raptors, the Andean Condor is threatened by habitat loss and human persecution throughout its range and thus globally classified as Near Threatened by the IUCN Red List [3]. It is included in Appendix I of the Convention on International Trade in Endangered Species of Wild Fauna and Flora [4] and in Appendix II of the Convention on the Conservation of Migratory Species of Wild Animals [5].

Population status of the Andean Condor is poorly known, although a total population of 6700 mature individuals in 2 540 000 km^2^ of extent of occurrence is estimated [6]. Individual population estimates from different countries partition rather differently into this global estimate. In Colombia and Venezuela, for example, less than 60 and 10 individuals have been estimated, respectively [7, 8]. In Peru fewer than 2500 individuals have been estimated [9], and a minimum population of 253 individuals has been in the central and austral Andes of Bolivia [10]. Argentina and Chile, meanwhile, harbor populations exceeding 3000 individuals each [11, 12, 13].

Ecuador is no exception to this situation, with surveys carried out between 1991 and 2012 having estimated less than 70 individuals in the country. For this reason, the species is currently categorized as Critically Endangered (CR) at the national level [14, 15, 16]. These estimations, however, were based on counts performed only in parts of the total Andean Condor habitat and are thus not representative of the entire distribution range of this species in Ecuador. In addition, differences in survey design and sampling effort preclude comparisons between the resultant estimates and limit conclusions on population trends. Next to small population size, several other processes (illegal hunting, competition with feral dogs, habitat loss and transformation, natural resource extraction and carrion poisoning threaten Andean Condor with high risk of extinction in Ecuador. Establishment of management actions for its conservation are therefore extremely urgent.

Current status of Andean Condor in Ecuador demands special attention because the lack of information on its autoecology hampers the correct assessment of conservation status and hinders the design and implementation of effective conservation actions. To address this situation, we assessed the conservation status of Andean Condor in Ecuador. The specific objectives of this study were i) to estimate the extent of occurrence and area of occupancy of Andean Condor, ii) to assess the effectiveness of natural protected areas in the conservation of the species, iii) to estimate the population size and iv) to evaluate the population viability of Andean Condor under different threat scenarios during the next 100 years.

## Materials and Methods

### Ethics statement

This work was carried out with support and permission of the Ministry of Environment of Ecuador for the project “Research and Ecological Monitoring of the Andean Condor in Ecuador” (research permit N° MAE-DNB-CM-2015-0010), approved by Dr. Lorena Tapia. Although field work involved an endangered and protected species, no animals were captured or sacrificed over the course of this study. The research permit N° MAE-DNB-CM-2015-0010 authorizes work with endangered species.

### Geographic distribution and Gap analysis

Andean Condor geographic distribution, in the form of extent of occurrence (EOO) and area of occupancy (AOO) [17], was estimated by means of ecological niche modeling. We used the maximum entropy method to generate a species distribution model (SDM) based seven environmental predictors with spatial resolution of 500 m (Table 1) and geographic coordinates of 60 roosting sites (used as presence records) obtained from satellite telemetry data of seven tagged Andean Condors [18].

**Table 1:** Environmental predictors used to model geographic distribution of Andean Condor (*Vultur gryphus*) in Ecuador.

Variable	Description
Bio_1	Annual mean temperature (WorldClim) [19]
Bio_12	Annual precipitation (WorldClim) [19]
Elevation	Ground height in relation to sea level (CGIAR-CSI) [20]
Slope	Ground inclination relative to the horizontal plane. Derived from Elevation
Aspect	The direction in which a slope faces. Derived from Elevation
Terrain roughness	Topographic index. Quotient of surface area/planimetric area. Derived from Elevation
Land cover	Categorical variable with land cover information (Terra MODIS MCD12Q1) [21]

The SDM model was generated in MaxEnt 3.3.3k [22]. For better model performance and complexity and to reduce miscalculation in estimated area of potential distribution, we tested different settings using a regularization multiplier (0–2) and several feature classes (linear, quadratic, product, threshold, hinge and combinations thereof) using the R package ENMeval [23] (https://www.R-project.org). The final setting in MaxEnt was selected using the Akaike Information Criterion [24].

MaxEnt final configuration included a regularization multiplier = 1.5; feature classes = Linear, Quadratic; convergence threshold = 10^−5^; and maximum iterations = 500. Model accuracy was evaluated using the area under the curve (AUC). AUC values above 0.8 were considered indicators of good accuracy. Ten replicates were performed and the SDM with the greatest value of entropy was selected as the final model. The final model was converted into a binary model using the minimum training presence threshold to estimate EOO and maximum training sensitivity plus specificity threshold to estimate AOO.

We performed a gap analysis [25] in order to evaluate Andean Condor conservation effectiveness (defined as extent of Andean Condor habitat available in existing conservation areas) of natural protected areas included in the “Sistema Nacional de Áreas Protegidas de Ecuador” (SNAP). For this analysis, we intersected polygons of EOO and AOO with those of the SNAP in ArcGis 10.1 [26] to estimate the extent (in km^2^) of EOO and AOO inside protected areas.

### Population size

Population sizes of Andean Condor were estimated in roosting sites surveyed simultaneously in the Andean region of Ecuador. A total of 163 observers positioned within a distance ≤ 2 km from the roosting site performed 840 hours of observation during September 29–30, 2015. Roosting sites were selected based on satellite telemetry data from seven tagged Andean Condors [18]. The criterion for selecting a roosting site included geographic coordinates of Andean Condor at 06:00 and 19:00 hours, frequencies of use of roosting sites ≥ 5 and distance among roosting sites ≥ 3 km. We also included roosting sites not detected with satellite telemetry based on information from SNAP park rangers and amateur birdwatchers.

**Fig 1:**
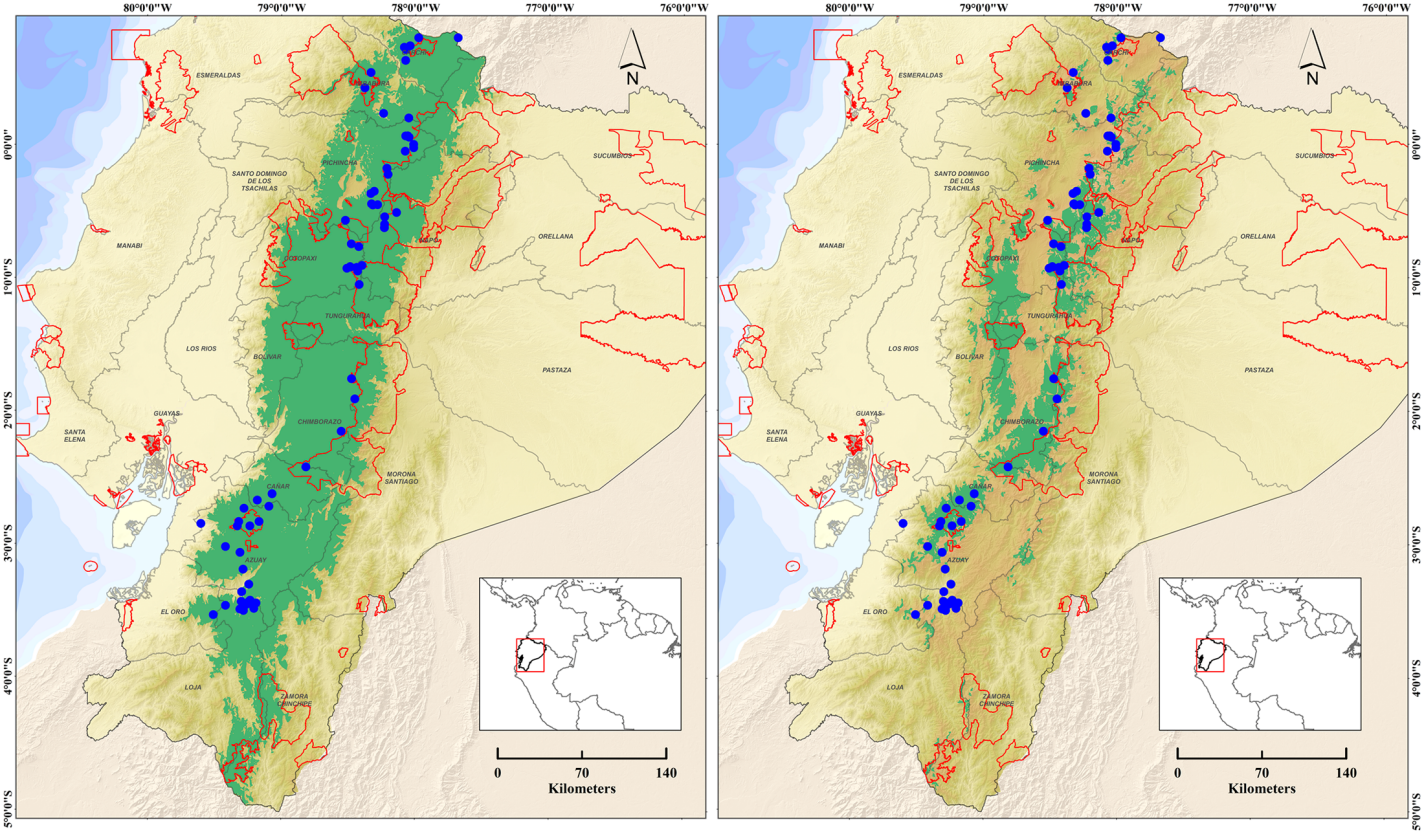
Geographic distribution of Andean Condor (*Vultur gryphus*) in Ecuador. Blue dots are roosting sites surveyed during September 29–30, 2015. Red polygons represent the National System of Protected Areas. Green areas represent extent of occurrence (left) and area of occupancy (right).

Seventy roosting sites (Fig 1) were surveyed between 15:30–18:30 and 06:00–09:00 hours on 29 and 30 September 2015, respectively. Observations were performed in 15-minutes intervals using binoculars (10 x 42) and spotting scopes (20–60 x 80). Absolute numbers, age and sex of observed Andean Condors were recorded using the following classifications: adult male, adult female, adult unsexed, subadult male, subadult female, subadult unsexed, juvenile male, juvenile female, juvenile unsexed and undetermined age and sex [10].

We estimated the numbers of individuals in each age class and sex, as well as the proportion of ages and sexes. To compare our results with similar previous studies on this species [10, 27, 28], subadults and juvenile condors were grouped into a single class denominated immature.

Because the assumptions of normality and homoscedasticity were not met, we performed a Welch’s *t*-test to evaluate the difference between absolute numbers of Andean condors observed during the afternoon of day 29 and the morning of day 30 and a Welch’s ANOVA to explore differences among time intervals [29]. Differences among age and sex classes were evaluated with a Chi-Squared test of independence [29] whereas a difference in the ratio of adults and immatures was tested with a Chi-Squared goodness-of-fit test [29].

### Population viability

We evaluated population viability using VORTEX V10, a program for modeling vertebrate population viability [30]. Demographic parameters (a total of 17, Table 2) used in VORTEX were obtained from the population size estimated in this study and data on reproductive biology from pertinent literature [1, 2, 31]. Vortex requires a carrying capacity estimation for fitting its predictions. This information is not available for the Andean Condor. Therefore, we extrapolated the estimated number of at least 10,000 individuals in 2,540,000 km^2^ of EOO [6] to the estimated EOO corresponding to the area of Ecuador.

**Table 2:** Demographic parameters used in the population viability analysis of Andean Condor (*Vultur gryphus*) in Ecuador.

Parameter	Value	Reference
Age of first offspring (years)	7	2
Maximum number of broods/year	1	1, 2
Maximum number of progeny/ year	1	1, 2
Sex ratio at birth in % ♂	50	This study
% adult females breeding	36	This study
Mortality from age 0 to 7 (%)	7.1	31
Annual mortality after age 7 (%)	7.1	31
Adult ♀	24	This study
Subadult ♀	7	This study
Juvenile ♀	5	This study
Adult ♂	22	This study
Subadult ♂	2	This study
Juvenile ♂	6	This study
Carrying capacity	195	This study

We modeled population viability for the next 100 years using 11 different scenarios. For each scenario, VORTEX estimated population size, probability of extinction, mean time to extinction and loss of genetic variability. We evaluated statistical significance of population size and genetic variability trends within a 100-year period by means of a linear regression [29]. In our basic scenario (scenario 1) there was no human persecution or habitat loss. Scenario 2 considered habitat loss in the Ecuadorian Andes at an annual rate of 0.8%, an estimation obtained from land cover maps of continental Ecuador from 1990 and 2014 [32, 33]. We modeled human persecution in scenario 3 using harvest as a surrogate of illegal hunting, a rate of one individual per year was applied, independent of age or sex [31]. Impacts of habitat loss and human persecution on extinction risk (scenario 4) were examined by combining parameters of scenarios 2 and 3. Increased habitat loss was considered in scenario 5, in which annual rate of habitat loss was doubled from 0.8% to 1.6%. In scenario 6, we evaluated extinction risk caused by cryptic hunting, defined as undetected illegal hunting. In this scenario, we doubled harvest from one to two individuals per year, again independent of age or sex. The effects of increased habitat loss (scenario 5) and cryptic hunting (scenarios 6) were studied by combining these two conditions in scenario 7. Scenario 8 explored the possible influence of habitat loss at an annual rate of 0.8% (scenario 2) and cryptic hunting (scenario 6). Finally, the consequences of increased habitat loss (scenario 5) and human persecution (scenario 3) were modeled in scenario 9.

In addition to the threat scenarios described above, we modeled population reinforcement as a conservation action using the rates of habitat loss and human persecution of scenario 4. Population reinforcement of two and four individuals was evaluated in scenario 10 and 11, respectively, using a male-to-female ratio of 1:1.

## Results

### Geographic distribution and Gap analysis

The SDM was accurate with an AUC value of 0.93. EOO and AOO of Andean Condor in Ecuador were 49,550 km^2^ and 14,106 km^2^, respectively (Fig 1). The altitudinal distribution varied from 600 to 6,280 meters above sea level (m.a.s.l.) for EOO and 1,080 to 6,280 m.a.s.l. for AOO. Gap analysis revealed that the representation of Andean Condor distribution within SNAP varied from 23% (EOO) to 34% (AOO).

### Population size

Andean Condors were observed in 38 (54%) of 70 roosting sites surveyed. The total number of Andean Condors was 93 individuals. Data corresponding to condors’ observation were fitted to a Poisson distribution. The estimated number of Andean Condors varied between 78 and 110 individuals (90% confidence intervals with Poisson distribution) and 94 and 102 individuals (90% confidence intervals with Gaussian distribution).

Of the total individuals observed, 14 (15%) were not fully identified by age and/or sex, and these were not included in the analysis of population structure. Table 3 shows the total number of Andean Condors by age classes and sex. Male-to-female sex ratio was 1:1.2, whereas the sex ratio, adult male-adult female and immature male-immature female were 1:1.09 and 1:1.5 respectively. However, these differences were not statistically significant (χ2 = 0.344; df = 1; *p* = 0.557). A greater number of adult condors (65%) in respect to immature (35%) was observed, yielding a significant ratio of 1:0.5 (χ2 = 7.911; df = 1; *p* = 0.004).

**Table 3:** Total number of Andean Condor (*Vultur gryphus*) observed during the survey in Ecuador (29–30 September 2015) and corresponding population size estimates (CI = confidence interval).

Age and Sex	Number observed	Population size estimate 90% CI Poisson distribution	Population size estimate 90% CI Gaussian distribution
Adult male	22	15–31	22–24
Adult female	24	17–34	24–26
Adult unsexed	6	3–12	6–7
Subadult male	2	0–6	-
Subadult female	7	3–12	7–8
Subadult unsexed	3	-	-
Juvenile male	6	1–8	6–7
Juvenile female	5	2–11	-
Juvenile unsexed	4	1–9	-
Age and sex not determined	14	8–22	14–15
Total	93	78–110	94–102

On September 29th a greater number of Andean condors was recorded in comparison to September 30th (63 *vs*. 45), yet this difference was not significant (*t* = 1.398; df = 59.584; *p* = 0.167). Differences in the number of condors observed among time intervals in both days were also non-significant (Afternoon: F_11, 174,5_ = 0.821; *p* = 0.619, Morning F_11, 174,7_ = 0.375; *p* = 0.964).

### Population viability

**Fig 2:**
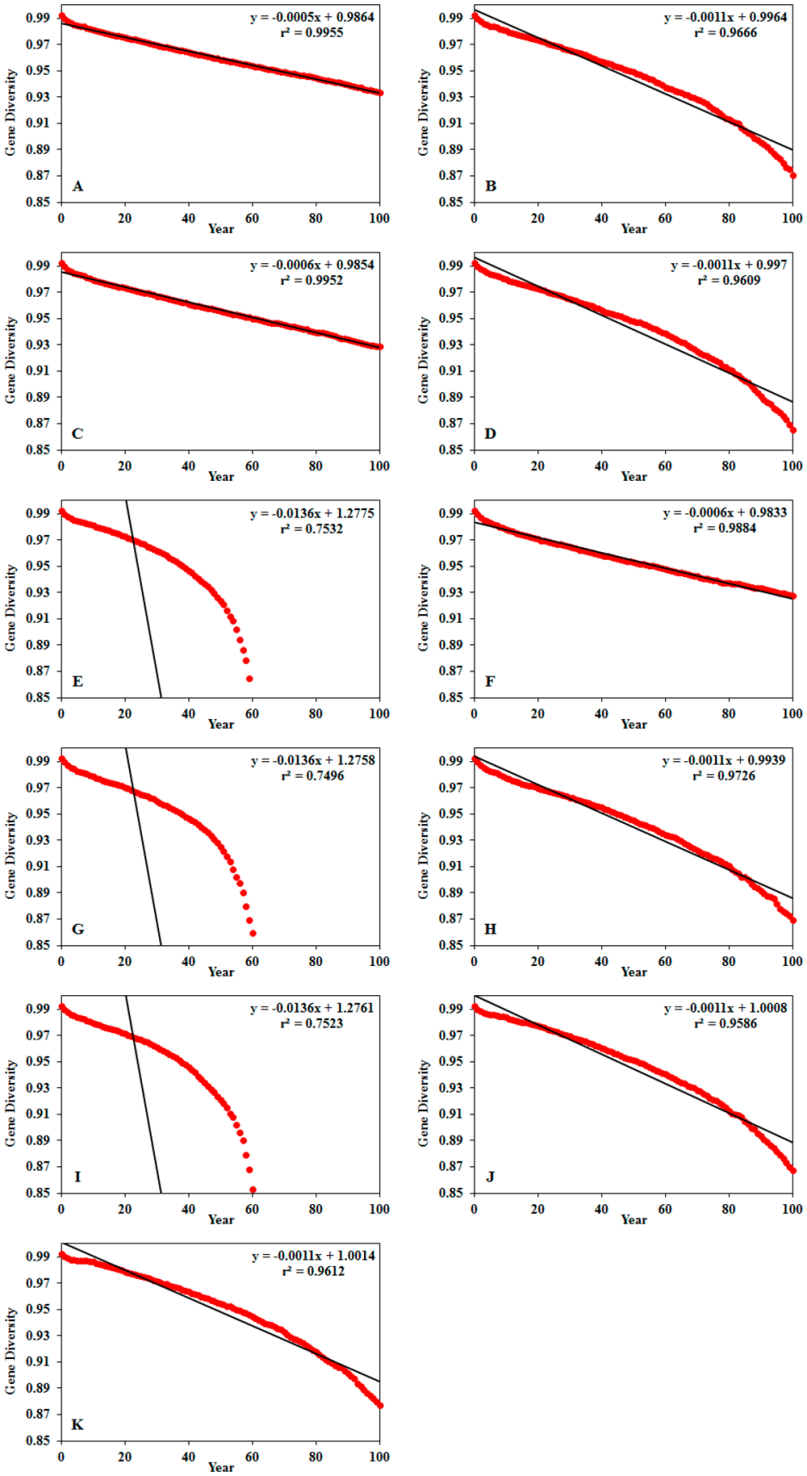
Loss of genetic variability in Andean Condor (*Vultur gryphus*) in Ecuador. Trends modeled in VORTEX V10. Genetic variability in vertical axis. Years in horizontal axis. A—Scenario 1 no human persecution or habitat loss, B—Scenario 2 annual habitat loss (-0.8%), C—Scenario 3 human persecution, D—Scenario 4 habitat loss and human persecution, E—Scenario 5 annual habitat loss (-1.6%), F—Scenario 6 cryptic hunting, G—Scenario 7 annual habitat loss (-1.6%) and cryptic hunting, H—Scenario 8 annual habitat loss (-0.8%) and cryptic hunting, I—Scenario 9 annual habitat loss (-1.6%) and human persecution, J—Scenario 10 population reinforcement two individuals/year, K—Scenario 11 population reinforcement four individuals/year. *p*-value was < 0.001 in all scenarios.

**Fig 3:**
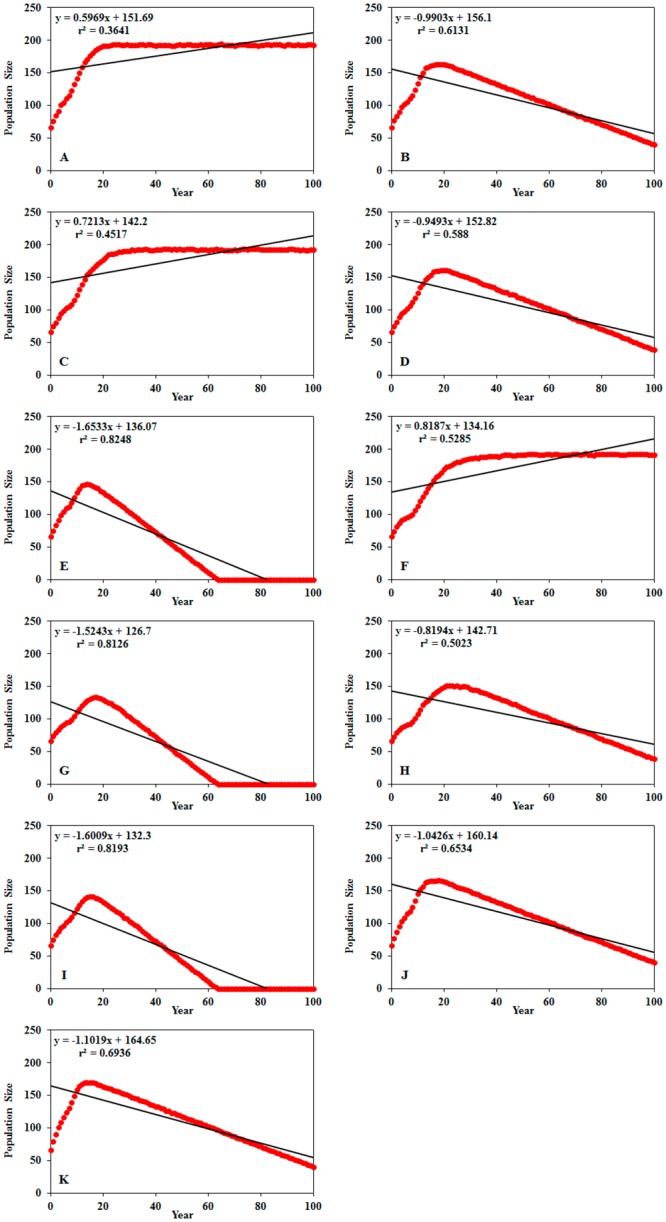
Population size trends of Andean Condor (*Vultur gryphus*) in Ecuador. Population trends were modeled in VORTEX V10. Population size in vertical axis. Years in horizontal axis. A—Scenario 1 no human persecution or habitat loss, B—Scenario 2 annual habitat loss (-0.8%), C—Scenario 3 human persecution, D—Scenario 4 annual habitat loss and human persecution, E—Scenario 5 annual habitat loss (-1.6%), F—Scenario 6 cryptic hunting, G—Scenario 7 annual habitat loss (-1.6%) and cryptic hunting, H—Scenario 8 annual habitat loss (-0.8%) and cryptic hunting, I—Scenario 9 annual habitat loss (-1.6%) and human persecution, J—Scenario 10 population reinforcement two individuals/year, K—Scenario 11 population reinforcement four individuals/year. *p*-value was < 0.001 in all scenarios.

In the modeled PVA scenarios, probability of extinction, mean time to extinction and minimum population size varied from zero to 100%, 63 years and 193 individuals, respectively (Table 4). In all scenarios, VORTEX predicted a loss of genetic variability relative to the first year of the 100-year period. Population size trends and loss of variability along the 100-year period were significant (*p* = 0.000) in all scenarios (Figs 2 and 3)

**Table 4:** Population viability analysis of Andean Condor (*Vultur gryphus*) in Ecuador at the end of 100-year period.

Scenario	Probability of extinction (%)	Population size	Mean time to extinction
1	0	193	0
2	0	40	0
3	0	193	0
4	0	39	0
5	100	0	63
6	0	192	0
7	100	0	63
8	0	38	0
9	100	0	63
10	0	40	0
11	0	40	0

**Fig 4:**
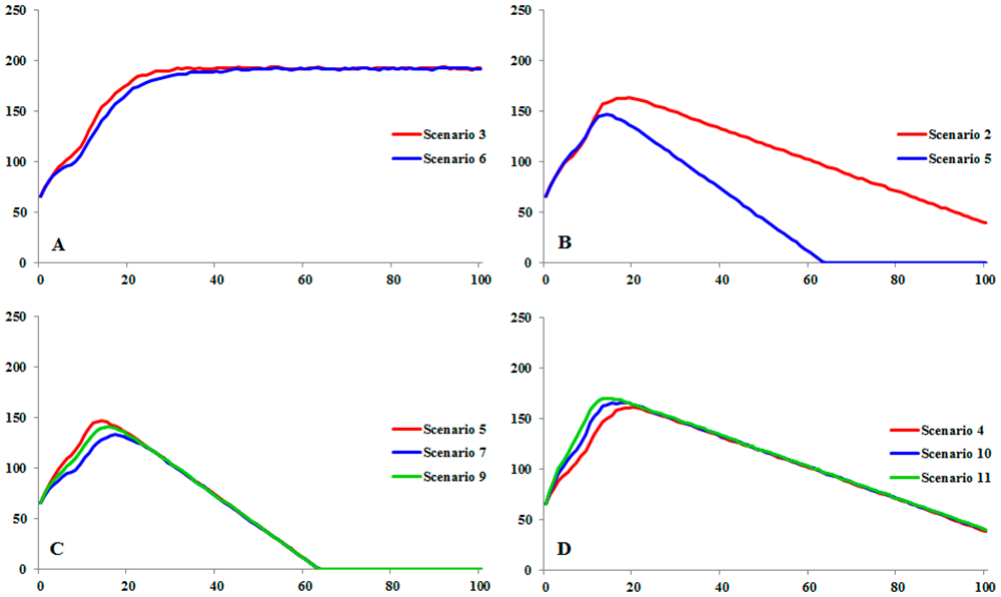
Comparison of population size trends of Andean Condor (*Vultur gryphus*) in Ecuador among scenarios with different threats and management conditions. Trends modeled in VORTEX V10. Population size in vertical axis. Years in horizontal axis.

Results of scenarios 3 and 6 (Fig 4A) suggested human persecution to have no negative impacts in Andean Condor population when compared to habitat loss (scenario 2 and 5, Fig 4B). Scenario 4, which corresponds to the current situation of Andean Condor in Ecuador, predicted maximum increase of 161 individuals in the Andean Condor population at the end of the 19 year period and a population reduction starting three years after the maximum increase if current threat conditions are maintained.

According to PVA results, habitat loss is the greatest threat to the conservation of Andean Condor populations in Ecuador; population size reduction in scenarios that included this threat began during the first 15 years. Although current habitat loss ratio (scenario 2) will not drive Andean Condor to extinction in the next 100 years *per se*, this situation would be different if this ratio increases (scenario 5) or is combined with human persecution (scenarios 4, 7, 8 and 9). In scenarios 5, 7 and9, decrease of population size began by the end of 15-year, 18-year and 17-year periods, respectively. In these scenarios, Andean Condor extinction was predicted within 63-years (Fig 4C).

Population reinforcement (scenarios 10 and 11) had no effects in the recovery of the Andean Condor population given the current population parameters of the species in Ecuador (scenario 4; Fig 4D). At the end of the 100-year period, probability of extinction, mean time to extinction and minimum population size values were the same in scenarios 4, 10 and 11 (see Table 4). Maximum population size reached 166 individuals by the end of a 15-year period for scenario 10 and 170 individuals in the last year of a 17-year period in scenario 11. Even though population decline began the following year; these values were close to those described above for scenario 4.

## Discussion

### Geographic distribution and Gap analysis

There have been no previous quantitative estimates of geographic distribution of Andean Condor in Ecuador. Although EOO and AOO are criteria used by red list authorities to assess species conservation status [17], past IUCN reports [14] do not include these estimates for Andean Condor in Ecuador. EOO reported in this study represents 1.9% of the estimated extant for South America [6].

Estimates of Andean Condor EOO and AOO are available in the literature for Colombia and Venezuela [7, 34]. These estimates vary between 33% and 71% and 46% and 75%, respectively, in relation to the estimates presented in this study. Differences in EOO and AOO values are linked to the environmental heterogeneity of each country which influences niche variables and dispersion dynamics regulating distribution range limits [34].

Results indicate that SNAP is protecting about 20% of the Andean Condor geographic distribution in Ecuador. This level of protection may be insufficient for highly mobile species such as the Andean Condor [35]. For less mobile species, protection of 10% to 70% of species habitat has been suggested as a biodiversity conservation goal [36], while at least 30% protection of the geographic distribution has been proposed for the species of interest [37].

### Population size

Since the beginning of the 1990s, Andean Condor population size in Ecuador has been estimated on several occasions. However, estimates differ in regard to geographic scope methods, sampling effort and reporting thereof, precluding significant comparison. For example, one estimate [38] cites 70 individuals without specification of geographic area and sampling effort. Surveys performed by means of photo-identification and simultaneous survey in central north of Ecuador reported a maximum of 27 individuals [39]. Ina survey completed in the northern section of the Ecuadorian Andes, a total of 54 individuals were recorded [40]. A minimum number of six individuals in Cajas National Park, in southern Ecuador, using three different census techniques [16].

Simultaneous census of condors in roosting sites avoids the double counting of individuals during survey times [13] and thus provides greater accuracy in estimating the number of Andean Condors in Ecuador. Although this census and others were performed simultaneously, it is not possible to determine population trends of Andean Condor due to differences in the numbers of roosting sites and sampling effort used in each survey.

The population estimate presented in this study includes values obtained from two different probability distributions. Data were first fitted to a Poisson distribution, yet given that this assumes the evaluated parameter to be discretely distributed, we proceeded to an estimation based on the Gaussian curve. The latter distribution is suitable for continuous variables and also provides more conservative results with smaller confidence intervals.

In contrast to results of other studies surveying roosting sites, our estimate is lower than reports by for some conservation priority areas in Peru (160–273 individuals [9, 41]) and central Argentina (246 individuals [13]). Population density estimates for Ecuador correspond to 0.18 individuals/100 km^2^ in Ecuador, a value substantially lower than those reported in central Argentina (3.89 individuals/100 km^2^). It is not possible to estimate population density of Andean Condor in Peru given that available estimates [41] do not specify study area extent. Differences in estimates for Ecuador, Peru and Argentina may be artefactual but also likely to be associated with environmental factors related to the availability of resources that directly affect the abundance of the species.

Population structure of Andean Condor has been the subject of previous research along its distribution range. In Ecuador, 65% of the population corresponds to adults with a sex ratio of 1:1.12, corresponding to an estimated26 potential breeding pairs. These results are consistent with those reported in northern Ecuador [28] and other areas of this species’ distribution [13, 27]. The adult-to-immature ratio found in this work (1:0.5) is similar to those of Argentina (1:0.46; [13]), Chile, (1:0.52; [42]) and Peru (1:0.35; [43]) and suggests a low rate of recruitment in the population. This could be explained by the low reproductive rate of the species, high natural mortality rate of immature individuals [13] or mortality caused by human activities. In Bolivia, the number of immature condors is greater than adults, and this ratio is used as an indicator of a reproductively healthy population [44, 10].

In contrast to male-skewed sex ratios found in other Andean Condor populations [45], sexual segregation was not observed in Ecuador. Probable causes for sexual segregation reported elsewhere might involve differences in habitat use and human persecution. Although our results cannot exclude sexual segregation in Andean Condor populations of Ecuador, they suggest that both males and females have the same probability of impacted by human persecution. This implies severe risk to population survival, considering the presence of only 26 breeding pairs in the entire country.

## Population Viability

The inferences derived from the population viability analysis have implications for Andean Condor management in Ecuador. This population tends to remain stable during the next 100 years if there is no additional habitat loss. Habitat loss is the main threat for Andean Condor conservation in Ecuador. All the scenarios that included this threat suggested population reductions in the first 25 years of simulation. These results suggest the need of new and updated land use management plans and policies. These policies and plans should be prioritized to the *páramo* ecosystems that constitute the main habitat of the Andean Condor in Ecuador.

The conservation status of *páramo* ecosystems in Ecuador has been compromised due to agricultural expansion into higher elevations, burning of grasslands, establishment of forest plantations and development of mining activities [46]. According to the land cover map of Ecuador [32, 33], certain vegetation cover classes (e. g. forest plantations) have increased in the last 24 years at an annual growth rate of 3%. This increase has no positive impact on the population of Andean Condor.

Although extinction is not forecasted within the next 100 years for the current disturbance scenario of Andean Condor in Ecuador, population size may be reduced below the current population estimate in the next six decades. The most pessimistic scenarios estimate onset of population size reduction and extinction to occur in the next 20 and 50 years, respectively.

Although inbreeding depression was not considered in the analyses because there is no data available for Andean Condor, all the scenarios showed a loss of genetic variability starting at the first year of the simulations and suggest the presence of an ongoing inbreeding process with a possible bottleneck effect. However, this speculation requires confirmation from population genetic research. Inbreeding depression is expected in small populations of large-sized vertebrates like the Andean Condor. Genetic variation is positively and negatively correlated to population size and body mass, respectively [47, 48]. This is particularly critical in closed populations where no gene flux and a small number of individuals exist. In our modeled scenarios, we assumed Andean Condor in Ecuador to comprise a closed population. Although GPS-tagged condors from Ecuador occasionally make brief visits (1–2 days) to southern Colombia, no dispersal movements from Ecuador to Peru have been recorded (Vargas, unpublished data). There are also a handful of cases in which captive-raised condors released in southern Colombia were recorded in northern Ecuador. Contact between populations in the northern Andes might prevent the loss of genetic variability.

The prediction of genetic drift in wild populations of Andean Condor in Ecuador, although hypothetical, should be considered as a warning for the genetic management of the population. Gene flow may be facilitated by translocations and population reinforcement with individuals from other populations. Although Andean Condor populations across the distribution range have low genetic variability, some degree of differentiation exists between northern and southern populations [48]. We encourage further research on this subject to improve the design and management of conservation actions such as translocation, reinforcement and *ex situ* management.

A captive-breeding program as a conservation action to reinforce the wild Andean Condor population has been developed in Ecuador since 2009 [49]. However, the number of captive-raised individuals to be released for improved species persistence has not been estimated yet. Population reinforcement of Andean Condor in Ecuador might not be effective under current conditions. Our results demonstrate that populations with and without reinforcement begin to decrease at the same time. Again, habitat loss is responsible for population decline in these scenarios. This study highlights the need to redirect efforts from captive breeding and population reinforcement to habitat conservation. Review of species status, including threat analysis, has been designated as the first step of the process to determine when *ex situ* management should be applied [50]. So far, this study constitutes the first systematic review of the conservation status of Andean Condor in Ecuador. Establishing a new legal conservation framework, we encourage environmental authorities and conservationists to redefine and prioritize conservation actions and policies for the conservation of Andean Condor in Ecuador: rates of habitat loss and human persecution must be reduced, in part by providing incentives to private ranch owners to protect Andean Condors, the species’ principal *páramo* habitat and its associated threatened biodiversity. Yet for the long-term conservation of the Andean Condor, awareness on the urgency of an integrated ecosystem management approach must be increased at all socio-economic levels.

## Supporting Information

S1 Appendix Organizations and volunteers that provided field assistance during the National Census of Andean Condors in Ecuador on September 29^th^ and 30^th^, 2015. (DOCX)
