# Supplementary material for: Andean Condor (Vultur gryphus) in Ecuador: Geographic Distribution, Population Size and Extinction Risk
Source: PLoS One. 2016 Mar 17;11(3):e0151827. doi: 10.1371/journal.pone.0151827 (PMC4795543; doi:10.1371/journal.pone.0151827)
Supplement: S1 Appendix — (DOCX) [file pone.0151827.s001.docx]

**S1 Appendix. Organizations and volunteers that provided field assistance during the National Census of Andean Condors in Ecuador on September 29^th^ and 30^th^, 2015.**

Ministerio del Ambiente del Ecuador, Wildlife Conservation Society Ecuador, The Peregrine Fund, Centro de Rescate Ilitío, Aves y Conservación, ETAPA, GAD Cuenca, GAD Nabón, GAD Oña, SIMBIOE, Universidad de Las Américas, Universidad del Azuay, Universidad San Francisco de Quito - Fondo Tueri, Zoológico Amaru, Fundación Zoológica del Ecuador, Fundación de Conservación Jocotoco, Fundación Galo Plaza Lasso. Parque Cóndor. Abraham Loaiza, Adrián Aguirre, Adrián Naveda-Rodríguez, Agustín Ordoñez, Alejandro Rosero, Alex Amón, Alfonzo Quezada, Álvaro Acosta, Ana Chacón, Andrés Marcayacta, Andrés Ortega, Ángel Palacios, Ángel Ushca, Anita Carrión, Augusto Granda, Benigno Carrión, Bolívar Amón, Byron Lumbana, Carlos Castro, Carlos Cuichan, Carlos Pasquel, Carlos Valdivieso, Cristina Regalado, Damián Ponce, David Arboleda, David Maldonado, Diego Cuichan, Diego Moreno, Diego Quispe, Diego Semanate, Dorys Minchala, Edison Araguillin, Edison Arboleda, Edison Moreno, Eduardo Obando, Edwin Marín Barba, Edwin Revelo, Edwin Taimal, Efrén Ramón, Elisa Levy, Eloy Salazar, Eric Guallpa, Ernesto Arbeláez, F Sánchez, F. Hernán Vargas, Fabián Cabrera, Fabián Méndez, Fanny Tello, Fausto Siavichay, Fernando Juela, Fernando León, Florencio Sucuzhagñay, Francisco Sornoza, Freddy Chisag, Fredy Velazco, Gabriela Montoya, Galo Zapata-Ríos, Geovanny Ascanta, Gerardo Cevallos, German Tenecota, Gia Brichetto, Gino Chiang, Giovanny Andrago, Gonzalo Gortaire, Guillermo Sangucho, Guillermo Z, Gumecindo H, Hari González, Hugo Arizaga, Ibeth Alarcón, Ignacio Flores, Ítalo Aimara, J. M. Gil-Sánchez, Jairo Guitarra, Javier Robayo, Javier Yépez, Jefferson García, Jhony Rivera, Jonathan Abad, José Cáceres José Luis Goyes, José Vieira, José Villa, Josué Arteaga, Juan C. Flores, Juan Cardozo, Juan Carlos Quezada, Juan Castillo, Juan Chillogallo, Juan Diego Molina, Juan Manuel Carrión, Juan Orellana, Juan Velastegui, Katherine Costa, Lorena Ramírez, Luis Calapi, Luis Carrasco, Luis Isquierdo, Luis Lucero, Luis Tambo, Luisa Machado, Manuel Gonzhi Tocuri, Manuel Gutiérrez, Manuela García, Marcelo Cordones, Marcelo Pantoja, Mariela Chauca, Mario Iglesias, Mauricio Iglesias, Max Araujo, Mayra Estrella, Miguel Acuñas, Miliar Ortiz, Milton Coronel, Nantan Inga, Omar Aguilar, Paola Asero, Patricio Cachumba, Patricio Escanta, Patricio Espín, Patricio Macas, Patricio Oña, Paul Aulestia, Paul Beltrán, Paul Ocampo, Paul Tito, Pedro Álvarez, Pedro Astudillo Webster, Quenny López, Rafael Ochoa, Ramiro Carpio, Ramón Pacheco, Ricardo Benavides, Robert Pontón, Roberto Palacios, Roberto Yánez, Rodolfo Avías, Rodrigo Toscano, Rolando Domínguez, Rolando Hipo, Román Criollo, Romel Fernández, Rubén Cueva, Ruth Muñiz López, Sandra Orellana, Sandra Paredes, Santiago Barros, Santiago Morales, Saúl Cáceres, Sebastian Kohn, Segundo Ruano, Shady Heredia, Silvia Cabrera, Simón Abad Méndez, Tania Aguirre, Tatiana Santander, Verónica Amoguimba, Víctor Utreras, Victoria Arbeláez, Vladimir Ushiña, Wilmer Neira, X Loyela, Xavier Laiguez, Ximena Jaya, Yann Potaufeu.
